# Survival and risk factor analysis in patients with septic arthritis: a retrospective study of 192 cases

**DOI:** 10.1186/s12879-024-10316-0

**Published:** 2025-03-18

**Authors:** Melanie Schindler, Lorenz Huber, Nike Walter, Josina Straub, Siegmund Lang, Dominik Szymski, Susanne Baertl, Dietmar Dammerer, Volker Alt, Markus Rupp

**Affiliations:** 1 Division of Orthopaedics and Traumatology, University Hospital Krems, Karl Landsteiner University of Health Sciences, Dr. Karl-Dorrek-Straße 30, Krems, 3500 Austria; 2 University for Continuing Education, Danube University Krems, Dr. Karl-Dorrek-Straße 30, Krems, 3500 Austria; 3 Department of Trauma Surgery, University Hospital Regensburg, Franz-Josef-Strauß-Allee 11, Regensburg, 93053 Germany; 4 Department for Psychosomatic Medicine, University Hospital Regensburg, Franz-Josef-Strauß-Allee 11, Regensburg, 93053 Germany; 5 Department of Trauma, Hand and Reconstructive Surgery, University hospital Gießen, Gießen, Germany

**Keywords:** Septic arthritis, Mortality, Comorbidities, Complication

## Abstract

**Background:**

Septic arthritis (SA) presents a complex clinical challenge, often resulting in significant morbidity and mortality. This study aimed to (1) assess overall mortality rates and (2) identify potential factors contributing to increased mortality risk in patients with SA.

**Methods:**

This retrospective study on SA patients treated at a German university hospital between January 1, 2011, and December 31, 2021. Patients were identified using International Classification of Diseases (ICD)-10 codes for septic arthritis, specifically “M00.-”. The study evaluated mortality rates and analyzed comorbidities, pathogens, and other potential risk factors. Kaplan–Meier survival curves and odds ratios (OR) were calculated to assess mortality risk.

**Results:**

In a cohort of 192 patients diagnosed with SA, 64 patients (33.3%) died during a mean follow-up period of 54.4 ± 42 months. The overall mortality rate was 17.5% at one year, 19.9% at two years, and 28.3% at five years. Patients aged 65 years or older, as well as those with arterial hypertension, congestive heart failure, chronic renal disease, chronic liver disease, malignancy, steroid use and immunosuppression showed significantly higher mortality rates (*p* < 0.05). Chronic renal disease (OR = 2.80), malignancy (OR = 3.40), and chronic heart failure (OR = 2.62) were identified as significant notably risk factors for mortality.

**Conclusion:**

This study highlights a notably high mortality rate among vulnerable patients with SA, particularly those with pre-existing comorbidities. Recognizing and addressing these risk factors early could improve patient outcomes. These results unterscore the need for close monitoring of SA patients, particularly those with chronic organ conditions, and timely intervention for sepsis to reduce mortality risk.

## Background

Septic arthritis (SA), an infection infiltrating joints, is commonly caused by bacterial invasion [1–3]. Septic arthritis typically presents as monoarticular but can also manifest as polyarticular [4]. Polyarticular involvement is noted in approximately 10–20% of patients [4]. The knee is more commonly affected compared to other joints [5, 6]. Despite experienced medical care, diagnosing septic arthritis can be challenging. An infection of a native joint can be classified into acute (< three weeks) and chronic (> three weeks) categories, collectively referred to as septic arthritis [7].

Immediate medical attention is essential as it is deemed a surgical emergency, requiring prompt assessment, diagnosis, and intervention. Timely recognition and treatment of SA play a crucial role in significantly reducing the risk of complications and mortality [2]. Conversely, delayed or insufficient treatment of septic arthritis may result in permanent joint damage, leading to subsequent disability, ultimately, mortality as the most severe consequence [5]. If an infection is confirmed or suspected, an early arthroscopic joint irrigation and joint debridement in terms of synovectomy should be performed [8].

SA is a relatively uncommon condition, with reported incidences ranging from four to twelve cases per 100,000 person-years (PY) [9–11]. The highest frequencies occur among individuals aged 55 years and older [6, 12]. Addressing the treatment of elderly patients, who are particularly vulnerable to these infections, is becoming increasingly important due to demographic development and the growing prevalence of comorbidities in an aging population [13].

The severity of septic arthritis is underscored by reported one-year mortality rates ranging from 11 to 19% [5, 14, 15]. Intriguingly, in contrast to diagnoses such as periprosthetic joint infection (PJI), where mortality rates are higher, the rates observed in our study are comparable to those in pyogenic spondylodiscitis but remain lower than those associated with major amputations [16–18]. Additionally, a substantial proportion of SA cases, estimated between 24% and 33%, encounter suboptimal functional outcomes [19–21].This joint infection is associated with a spectrum of established risk factors, including rheumatoid arthritis, diabetes mellitus, hemodialysis, intravenous drug use, alcohol dependency, intra-articular steroid injections, prior joint surgeries, cutaneous ulcers, and skin infections [14] .This study elucidates the complex spectrum of SA and underscores the importance of early detection and tailored interventions to reduce significant mortality rates and complications. Additionally, it highlights the necessity of comprehensive risk assessment, considering patient-specific factors and microbial spectra, to optimize treatment strategies and improve long-term outcomes.

The primary objectives of this study were: (1), to scrutinize the mortality rates of individuals affected by SA, stratified based on accompanying comorbidities, and (2), identify potential risk factors contributing to mortality among patients dealing with SA.

## Methods

This retrospective analysis involved patients aged 18 years or older receiving treatment for SA at a German university hospital. Patients were identified through the International Classification of Diseases (ICD)−10 codes for “M00.-, septic arthritis” within the period spanning from January 01, 2011 to December, 31 2021.

To verify diagnoses, patients’ medical records, surgical reports, laboratory findings, and both microbiological and histopathological records were examined. The patients exhibited pain, swelling, redness, or warmth in the affected joint, underwent joint aspiration with an increased cell count, or showed abnormalities in the affected joint on PET CT. Demographic data including sex, age, and body mass index (BMI) at the time of surgery and specific SA details, such as the affected joint and identified pathogens, were retrospectively assessed via electronic health records. Obesity was defined as a BMI ≥ 25 kg/m². Comorbidities were evaluated using the Charlson Comorbidity Index (CCI) [22]. To perform subgroup analyses, we divided the causes of septic arthritis into two categories: community-acquired septic arthritis (CASA) and healthcare-associated septic arthritis (HASA), as defined previously. This concept is adapted from the approach used in studies focusing on vertebral osteomyelitis (VO) [23]. Healthcare- associated VA (HAVO) was described as the development of symptoms occurring at least one-month post-hospitalization, within six months after hospital discharge, or subsequent to outpatient interventions within the preceding six months [24]. Specifically, HAVO, typically characterized by bloodstream infections involving low-virulence pathogens, manifests as an insidious disease course with prolonged diagnostic timelines and higher morbidity and mortality rates [24]. A minimum follow-up period of 24 months was established, during which all patients were contacted via telephone for clinical review.

In this study, severe infection or sepsis was identified by meeting the criteria of Systemic Inflammatory Response Syndrome (SIRS). According to this definition, a diagnosis of SIRS was established when a patient met at least two of the following criteria: (1) abnormal body temperature, indicated by a fever exceeding 38.3 °C (100.9 °F) or hypothermia below 36.0 °C (96.8 °F); (2) tachycardia, with a heart rate exceeding 90 beats per minute; (3) increased respiratory rate, characterized by over 20 breaths per minute or a decreased partial pressure of carbon dioxide in arterial blood (PaCO2) below 32 mmHg; (4) abnormal white blood cell count, demonstrated by either an elevation (> 12,000 cells/mm³) or a reduction in total white blood cell count (< 4,000 cells/mm³), or the presence of more than 10% immature forms (band forms) in the blood [25].

### Statistical analysis

SPSS Statistics version 28.0 (IBM SPSS Inc, Chicago, IL, USA) was used to analyze the data. Descriptive statistics were computed for all variables, demonstrating continuous variables through mean values and standard deviations. Each variable was individually examined to assess its potential role as a risk factor linked to in-hospital mortality among patients diagnosed with VO. The dataset encompassed various patient outcomes, including instances with and without in-hospital deaths.

To evaluate recurrence-free survival, Kaplan-Meier analysis was applied using follow-ups as endpoints. The ANOVA test was employed to establish the statistical significance of the relationship between each parameter and in-hospital mortality. A multifactorial analysis was conducted to examine the relationship between significant risk factors and mortality. Odds ratios (OR) with lower and upper 95% Confidence Intervals (CI). were calculated for different comorbidities and complications.

The strength of the statistical connection between exposure to specific factors and in-hospital mortality was using the odds ratio (OR). An OR less than 1.0 suggested a negative association, indicating reduced odds of in-hospital mortality with exposure to that variable. Conversely, an OR greater than 1.0 suggested a positive association, implying higher odds of the defined outcome with exposure.

To explore the relationship between in-hospital mortality and each variable, a Chi-square test or correlation analysis of independence was executed. The level of statistical significance was established as *p* < 0.05.

### Ethical considerations

The Ethics Committee at the University Hospital Regensburg granted approval (reference number 20-1681_1-104). This research adhered to the principles outlined in the Declaration of Helsinki, with all participants providing informed consent.

## Results

The study evaluated 192 patients for SA, included 135 men and 57 women. These participants had an average age of 62.0 ± 15 years, ranging from 19 to 90 years. The mean BMI was 28.8 ± 7 kg/m². The mean CCI was recorded as 2.1 ± 2. Most patients (50.5%) had an American Society of Anesthesiologists (ASA) score of III, 22.4% had an ASA score of II, 16.7% had an ASA score of IV, 9.9% had an ASA score of I and 0.5% V (Table 1).

**Table 1:** Demographic data

	All *n* = 192	Deceased *n* = 64
**Mean Age (a)**	62.0 ± 15	69.8 ± 13
Age ≥ 65a	85 (44.3%)	41 (64.1%)
**Sex**		
Male	135 (70.3%)	46 (71.9%)
Female	57 (29.7%)	18 (28.1%)
**Mean BMI** (kg/m²)	28.8 ± 7	28.4 ± 7
**Mean CCI**	2.10 ± 2	3.4 ± 3
**ASA**		
I	19 (9.9%)	2 (3.1%)
II	43 (22.4%)	5 (7.8%)
III	97 (50.5%)	41 (64.1%)
IV	32 (16.7%)	16 (25.0%)
**Mean lenght of hospital stay** (days)	28.3 ± 23	32.1 ± 26
**Mean ICU admission (days)**	14.7 ± 18	12.9 ± 17
**Location of SA**		
Knee	98 (51%)	31 (48.4%)
Shoulder	35 (18.2%)	17 (26.6%)
Ankle	26 (13.5%)	6 (9.4%)
Hip	20 (10.4%)	7 (10.9%)
Hand	6 (3.1%)	1 (1.6%)
Elbow	4 (2.1%)	1 (1.6%)
Sternoclavicular	3 (1.6%)	1 (1.6%)

The mean hospital stay was 28.3 ± 23 days (2–146) (Table 1). In the study, half of the patients underwent open operative treatment, while the other 50% received arthroscopic intervention. Sixty-six patients, accounting for 34.4%, had undergone previous surgery on the infected joint. It was observed that 84.4% (*n* = 162) of cases presented with a monoarticular involvement, whereas 15.6% (*n* = 30) exhibited a polyarticular SA.

The etiological distribution of SA in the present study revealed diverse origins. The majority of cases were attributed to hematogenous sources, accounting for 62.0% (*n* = 119). Concomitant chronic osteomyelitis was diagnosed, accounting for 7.3% of the cases (*n* = 14) SA occurred in 16 cases following arthroscopy in this joint, whereas SA after previous joint injections accounted for 2.1% (*n* = 4). Implant-related cases categorized represented 7.8% of instances (*n* = 15). Posttraumatic reasons were identified in 9.4% of cases (*n* = 18), with the remaining cases classified under the “other” category at 9.4% (*n* = 18).

Pathogens were identified, with *Staphylococcus aureus (S. aureus)* (*n* = 60; 31.3%), *Staphylococcus epidermidis (S. epidermidis)* (*n* = 11; 5.7%) and other pathogens’ (*n* = 11; 5.7%) being the most common. *S. aureus* significantly presented more frequently as monoarticular arthritis (46 vs. 14, *p* = 0.047), and the two methicillin-resistant *S. aureus (MRSA)* cases exclusively exhibit polyarticular involvement. There was no observed correlation between a specific pathogen and increased mortality rates.

The primary foci were identified through a thorough focus search, including dental examination, chest X-ray, abdominal ultrasound, urine diagnostics, and, if necessary, further extended by a PET CT scan. Skin and subcutaneous tissue infections were the predominant focus in 24.5% of cases (Table 2).

**Table 2:** Primary infection foci

Infection type	*n* (%)
Skin and subcutaneous tissue	47 (24.5%)
Pneumogenic	30 (15.6%)
Urinary tract	18 (9.4%)
Prosthetic joints	17 (8.9%)
Intraabdominal	14 (7.3%)
Oral/dental	9 (4.7%)

### The 5-year survival of patients with SA

Throughout the study period, 64 patients (33.3%) died. Conversely, the 5-year survival rate was 71.7%. Notably, for 9 individuals (5%), the date of death remains uncertain. The mean duration of follow-up was 54.4 ± 42 months, spanning from 0 to 149 months.

**Fig. 1:**
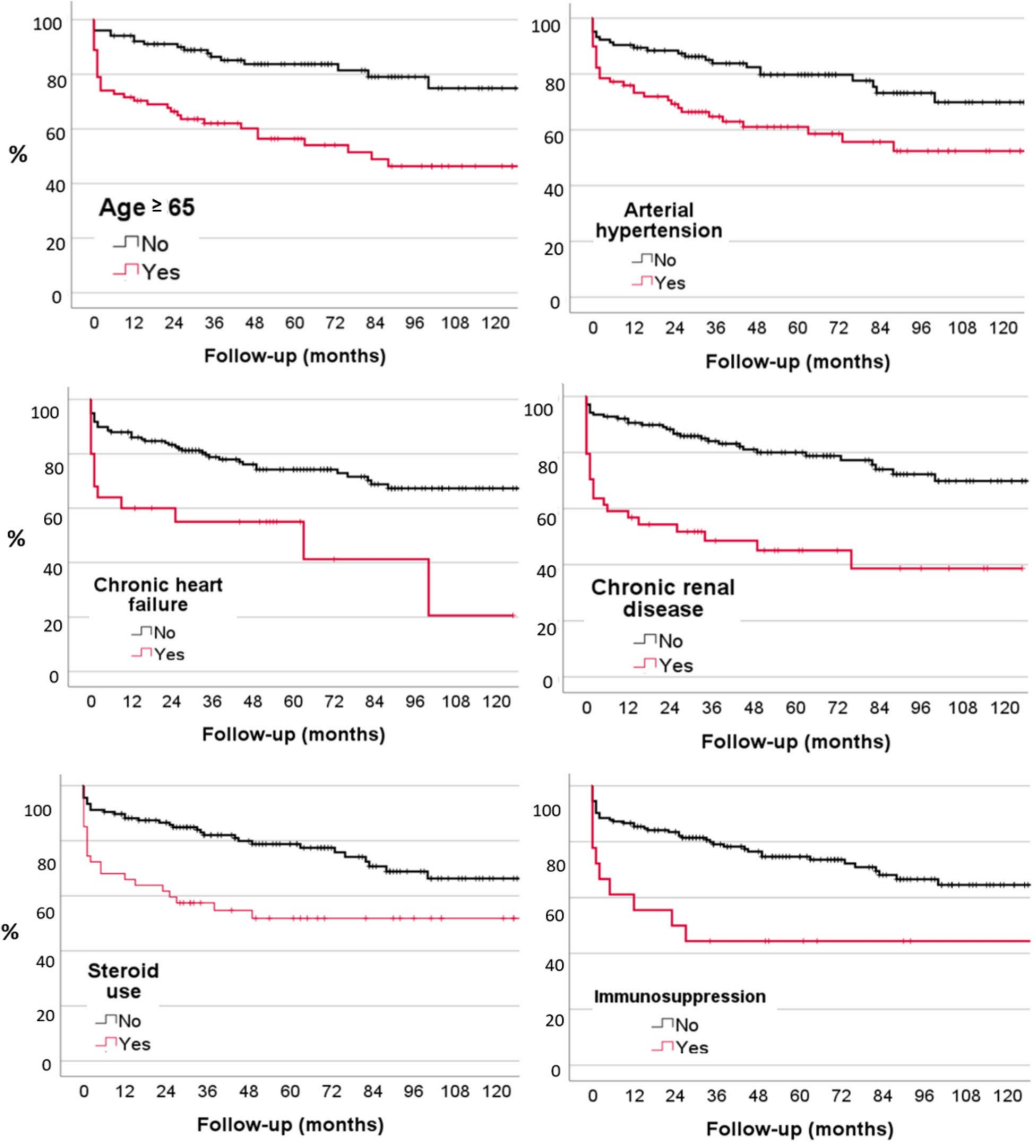
Kaplan-Meier probability plots of mortality related to age ≥ 65, arterial hypertension, chronic heart failure, chronic renal disease, steroid and immunosuppression use

The overall mortality was 17.5% at one year, 19.9% at two years and 28.3% at five years. Out of the total sample size of 13 patients (20.3%) died within 30 days (Fig. 1).

### Risk factors for mortality of SA patients

Patients who presented with age 65 or older (45.7%; *p* < 0.001), arterial hypertension (43.2%; *p* = 0.003), chronic heart failure (13.7%; *p* = 0.001), chronic renal disease (7.1%; *p* < 0.001), chronic liver disease (10.3%; *p* = 0.013), peripheral vascular disease (11.5%; *p* = 0.026), malignancy (13.7%; *p* < 0.001), steroid use (25.7%; *p* < 0.001), immunosuppression (10.0%; *p* = 0.003), intraabdominal infection (7.1%; *p* = 0.049), sepsis (24.2%; *p* < 0.001), septic shock (13.7%; *p* = 0.049) and ICU admission (30.6%; *p* < 0.001) demonstrated a significant higher rate of mortality than patients without these conditions (Table 3). After performing a multivariate analysis of the statistically significant risk factors for mortality, the following remained significant: age > 65 years (*p* < 0.001), arterial hypertension (*p* = 0.003), chronic renal disease (*p* < 0.001), chronic heart failure (*p* = 0.003), chronic liver disease (*p* = 0.020), malignancy (*p* < 0.001), steroid use (*p* = 0.006), immunosuppression (*p* = 0.015), sepsis (*p* < 0.001), septic shock (*p* < 0.001), and ICU admission (*p* < 0.001). Peripheral vascular disease and intra-abdominal infection did not show statistical significance. Otherwise, sex, previous hospitalization, other comorbidities such as diabetes mellitus, rheumatological disease, were not associated with an elevated mortality rate during the follow-up.

**Table 3:** Number of comorbidities and mortality

Comorbidities	*n* (%)	1-year mortality	2-year mortality	5-year mortality
Age ≥ 65a	81 (44.3%)	29.7%	33.6%	43.6%
Arterial hypertension	32 (40.5%)	24.1%	30.8%	39.0%
Chronic heart failure	13 (52.0%)	40.0%	40.0%	45.0%
Chronic renal disease	24 (54.5%)	43.2%	45.7%	54.9%
Chronic liver disease	7 (53.8%)	30.8%	48.7%	61.5%
Peripheral vascular disease	10 (47.6%)	33.3%	38.1%	42.9%
Malignancy	15 (60%)	37.0%	46.0%%	65.7%
Steroid use	22 (46.8%)	34.0%	38.3%	48.2%
Immunosuppression	10 (55.6%)	44.4%	50.0%	55.6%
Intraabdominal infection	6 (46.2%)	38.5%	47.3%	47.3%
Sepsis	23 (52.3%)	40.9%	40.9%	51.4%
Septic shock	16 (64.0%)	60.0%	60.0%	65.0%
ICU	28 (50.0%)	39.4%	41.3%	48.0%

**Fig. 2:**
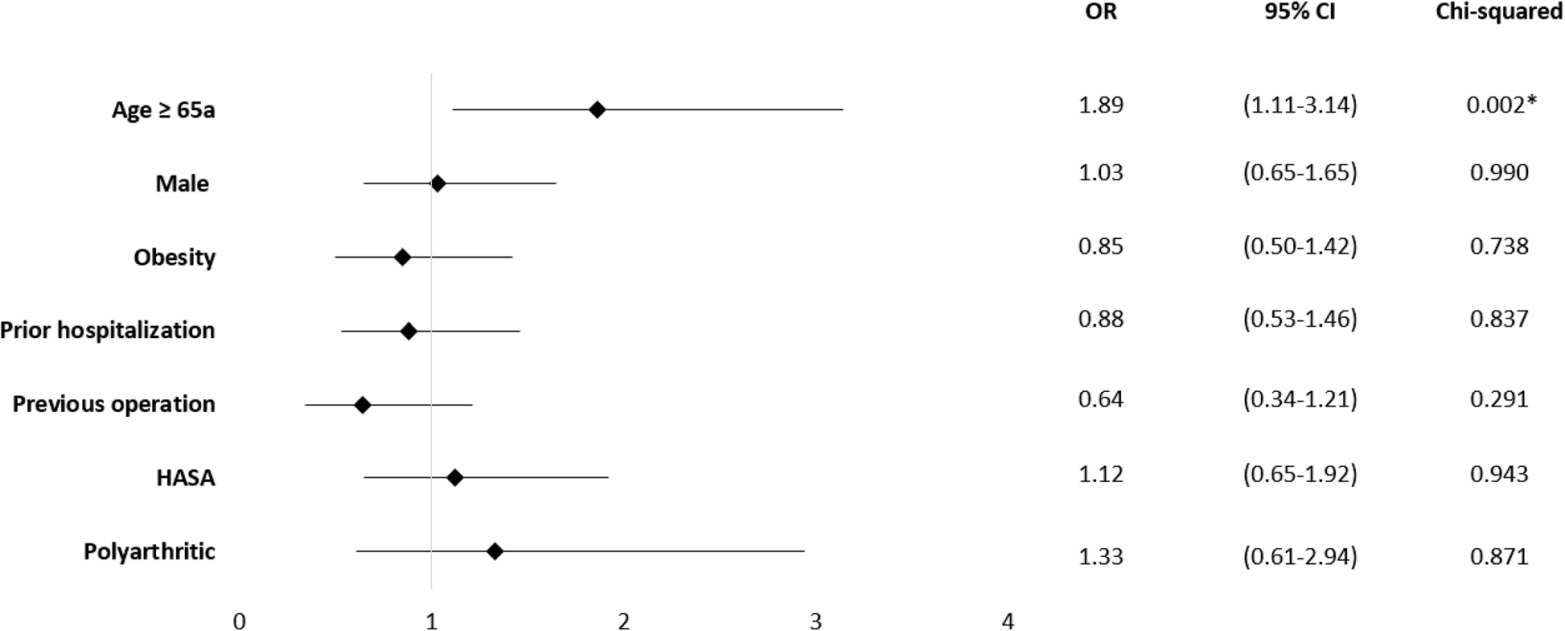
Mortality odds ratio for epidemiological and environmental factors

Age 65 years or older (OR = 1.86; 95% CI 1.11 to 3.14; *p* = 0.002) was significantly associated with mortality (Fig. 2). Based on the patient records, 81 cases were classified as HASA (42.2%), while 111 cases (57.8%) were categorized as CASA. Neither HASA nor CASA cases demonstrated an increased in-hospital mortality rate (Fig. 2).

**Fig. 3:**
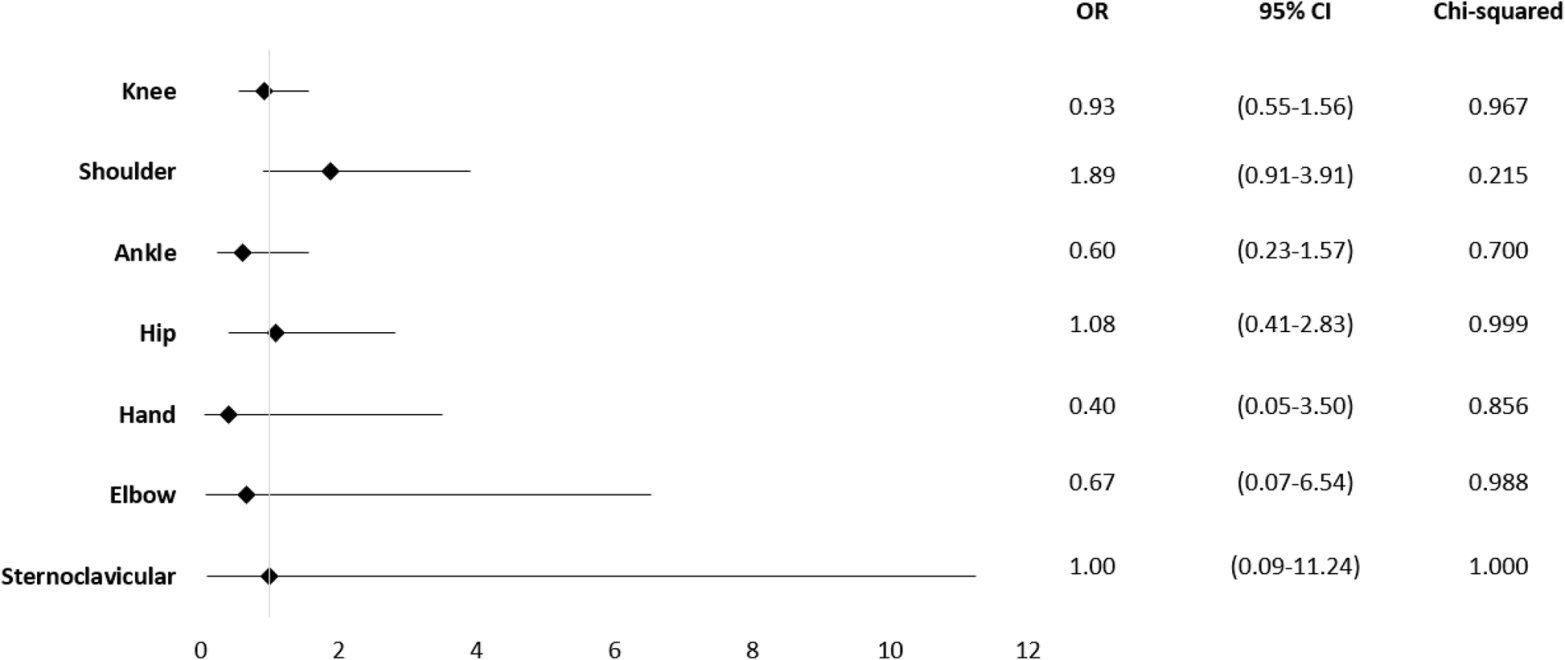
Mortality odds ratio for affected joints

The shoulder was the sole joint affected by a risk factor associated with increased mortality, without statistical significance (OR = 1.89; 95% CI 0.91 to 3.91; *p* = 0.215) (Fig. 3).

**Fig. 4:**
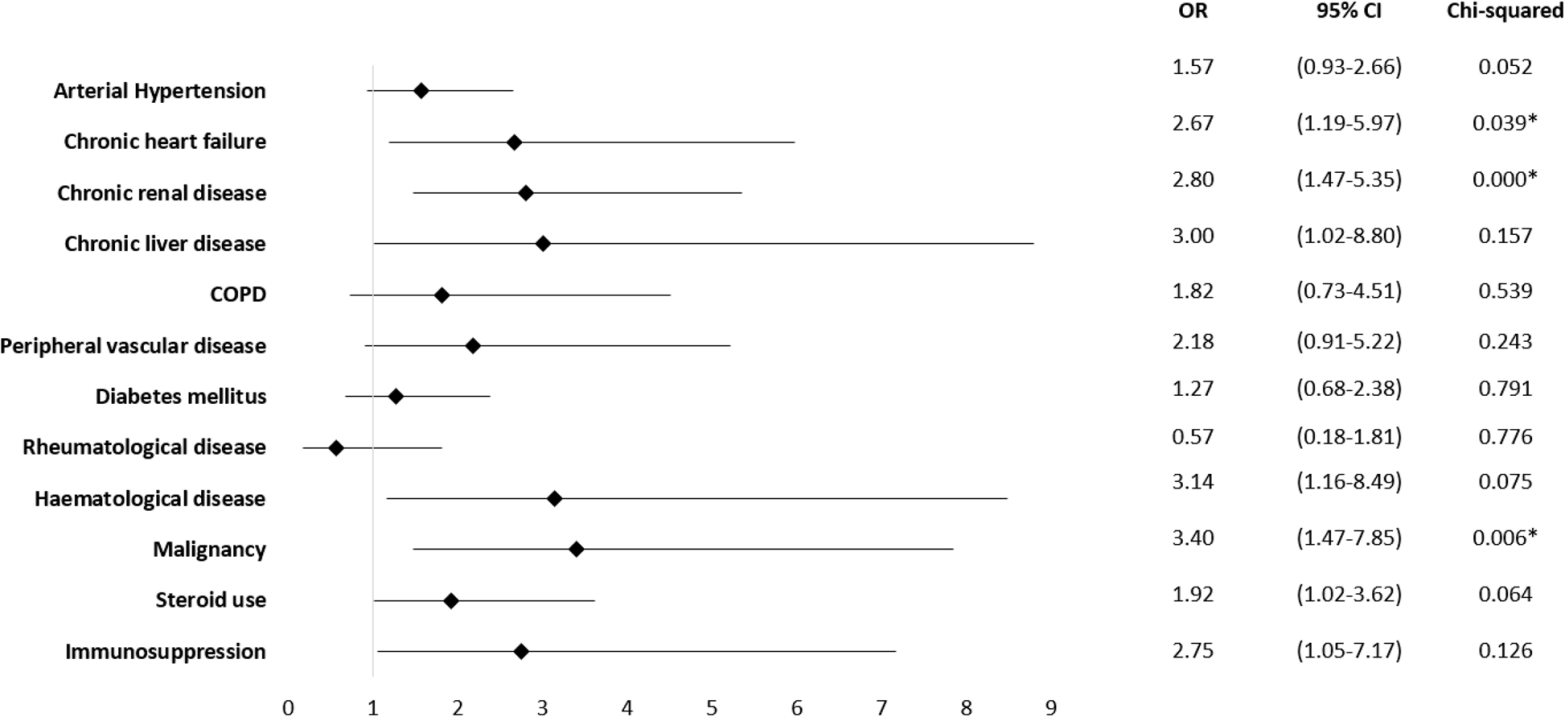
Mortality odds ratio for comorbidities

Chronic renal disease was identified as a risk factor (OR = 2.80; 95% CI 1.47 to 5.35; *p* = 0.000), malignancy (OR = 3.40; 95% CI 1.47 to 7.85; *p* = 0.006), as well as chronic heart failure (OR = 2.62; 95% CI 1.19 to 5.97; *p* = 0.039) (Fig. 4).

There was a statistically significant correlation found between mortality and CCI (*ρ*=−0.421; *p* < 0.001). The ASA score of the 64 those who passed away, was significantly elevated, with a mean of 3.11 compared to 2.58 (*p* < 0.001).

Sepsis (OR = 2.48; 95% CI 1.29 to 4.74; *p* = 0.004), septic shock (OR = 4.00; 95% CI 1.70 to 9.40; *p* = 0.001) and multiorgan failure (OR = 4.75; 95% CI 1.97 to 11.44; *p* < 0.001) were significantly associated with higher mortality.

## Discussion

The present case series demonstrated that SA is linked with a high rate of 30-days mortality and a substantial 5-year mortality rate. Regarding outcomes, the 5-year survival rate was 71.7%, with an overall mortality of 28.3% at five years. Patients with age over 65 years (45.7%), arterial hypertension (43.2%), chronic heart failure (13.7%), chronic renal disease (7.1%), chronic liver disease (10.3%), peripheral vascular disease (11.5%), malignancy (13.7%), steroid use (25.7%), immunosuppression (10.0%), intraabdominal infection (7.1%;), sepsis (24.2%), septic shock (13.7%) and ICU admission (30.6%) had notably higher mortality rates.

### The 5-year survival of patients with SA

The overall mortality was 7.1% at one year, 13.7% at two years and 23% at five years. Patients with septic arthritis and concomitant comorbidities were associated with increased mortality. Being 65 years or older (OR = 1.86), having chronic kidney disease (OR = 2.80) and experiencing congestive heart failure (OR = 2.62) were identified as a significant risk factors for a higher risk of earlier mortality. Additionally, the knee joint was the sole joint affected by a risk factor for a higher mortality (OR = 2.93).

The mortality rate found in this study is in line with the rates reported in previous research. Mortality associated with SA has exhibited variability, ranging between 11% and 19%, as indicated by several studies [5, 14, 15, 26]. Notably, a smaller prospective study reported an 11% mortality rate [14]. Maneiro et al. identified a one-year mortality rate of 16.6% following the diagnosis [26]. Conversely, Huang et al., in a national cohort study involving 31.491 SA patients, reported mortality rates of 4.3%, 8.6%, and 16.4% at 30 days, 90 days, and one year, respectively [15]. Another study involving 543 patients revealed a 90-day mortality rate of 5% [9]. These variations underscore the nuanced nature of mortality outcomes in SA and highlight the importance of contextualizing findings within the broader landscape of existing research. Possible patient factors that vary, as well as microbial spectra and potentially differing healthcare structures may also contribute to these differences.

### Risk factors for mortality of SA patients

In our cohort study, patients over the age of 65 years exhibited a considerable mortality rate of 45.7% (*p* < 0.001), highlighting the vulnerability of this age group. Furthermore, conditions such as arterial hypertension (43.2%; *p* = 0.003), chronic heart failure (13.7%; *p* = 0.001), chronic renal disease (7.1%; *p* < 0.001), chronic liver disease (10.3%; *p* = 0.013), peripheral vascular disease (11.5%; *p* = 0.026), malignancy (13.7%; *p* < 0.001), steroid use (25.7%; *p* < 0.001), immunosuppression (10.0%; *p* = 0.003), intraabdominal infection (7.1%; *p* = 0.049), sepsis (24.2%; *p* < 0.001), septic shock (13.7%; *p* = 0.049), and ICU admission (30.6%; *p* < 0.001) were significantly associated with increased mortality rates compared to patients without these conditions (Table 1).

Conversely, factors such as sex, previous hospitalization, and other comorbidities such as diabetes mellitus and rheumatological disease did not exhibit a statistically significant association with elevated mortality during the follow-up period. Notably, a significant correlation was identified between mortality and the CCI (*ρ*=−0.421; *p* < 0.001), emphasizing the importance of considering overall health status in prognostic assessments. Additionally, the ASA score among the 64 deceased individuals was found to be significantly higher, underscoring its potential as a predictive factor for adverse outcomes. This comprehensive analysis sheds light on the intricate interplay of various clinical parameters and their impact on mortality, providing valuable insights for risk stratification and patient management strategies.

Expanding our understanding of mortality in SA, existing literature indicates a 90-day mortality rate of 7%, escalating to 22–69% in patients aged 80 and older [27]. In other studies comorbidities such as diabetes mellitus, rheumatoid arthritis, bacteremia, and low creatinine clearance are also linked to increased mortality [19]. Specifically, patients with rheumatoid arthritis experiencing joint flare-ups are identified as particularly high-risk individuals [28, 29].

Significant prognostic factors identified in the literature include increasing age (*p* < 0.001), female sex (*p* = 0.046), higher CCI scores (*p* < 0.001), and lacking private insurance [30]. Yeh et al.‘s study on 52 dialysis patients with SA revealed tunneled cuffed catheter and fever as predictors of positive blood culture, with tunneled cuffed catheter being a predictor of in-hospital mortality [31]. A study examining SA in emergency departments in the United States revealed that the female sex, urban residence, treatment at a metropolitan teaching hospital, and the presence of medical comorbidities including diabetes mellitus, hyperlipidemia, hypertension, chronic obstructive pulmonary disease, coronary heart disease, gout, osteoarthritis, renal failure, and heart failure were associated with an elevated probability of hospitalization [32].

In line with prior investigations, our study recognized a similar pattern, with the knee emerging as the most frequently affected joint, closely followed by the shoulder. Specifically, 98 cases (51%) were related to the knee. A other study of 491 patients with SA, the knee emerged as the most frequently affected site, constituting 50.1% of cases, followed by the hip (14.4%), other anatomical locations (26.8%), the shoulder (5.5%), and involvement in multiple sites (1.2%) [15]. These findings align with current literature, emphasizing the significant prevalence of knee involvement [30, 33–35]. The richness of literature on native knee arthritis supports our findings, as it is recognized as the most commonly affected joint [1, 36–40].

Supporting the typical presentation, our study affirmed the predominant pattern of intra-articular infection, primarily characterized by monoarticular involvement. In alignment with established literature, our investigation also identified a congruent pattern, with approximately 20% of cases exhibiting participation in multiple joints, denoted as oligoarticular [9, 29].

In managing SA cases, our study employed arthroscopic or open surgical lavage in each case of SA. Several studies have conducted comparisons between operative intervention and medical therapy [41–43]. In one study, patients received empirical intravenous therapy with cloxacillin and ceftriaxone based on culture results, with treatment adjusted according to microbial sensitivity [43]. Meanwhile, another study assessed treatment failure after 7 days, with surgical treatment defined as a need for a second surgery [41]. Notably, in contrast to a study of Mabille et al. about knee and hip arthritis [41], our observed median duration of hospitalization was a little bit higher, specifically 28.3 ± 23 days, with extremes ranging from 2 to 146 days. Mabille et al. found that the median duration of hospitalization was significantly higher in the surgical group compared to the medical group (33.5 vs. 21 days) [41]. This difference was primarily attributed to post-intervention follow-up, particularly the extended rehabilitation period in a rehabilitation center within the surgical group. These findings confirm data published by Ravindran et al., emphasizing the impact of surgical intervention on post-treatment care and rehabilitation needs [42]. Timely intervention is imperative, encompassing the initiation of antibiotics alongside surgical lavage and debridement [44]. Arthroscopic methodologies have demonstrated comparable efficacy to conventional open techniques, offering the supplementary advantages of shorter hospitalization periods and enhanced postoperative recuperation [1, 6, 38, 45–47]. The decision to utilize an arthroscopic approach is based on the discretion of the treating physician, with additional advantages including minimally invasive procedures, wound healing, functional outcomes, and reduced blood loss.

Prevention is crucial in managing septic arthritis, as early intervention significantly reduces the risk of complications. Our institution follows German guidelines for the prevention and treatment of septic arthritis, including prompt antibiotic therapy and surgical intervention when necessary. Compliance with these guidelines is regularly monitored, suggesting that the findings may be applicable to other centers in Germany.

The etiology of SA often presents a diagnostic challenge, with a significant proportion of cases lacking an identified causative bacterial organism. However, when a pathogenic organism is identified, *S. aureus* emerges as the most prevalent, constituting 53% of the cases [9, 30, 31, 34, 38, 41]. In our study, consistent with existing literature, *S. aureus* was the predominant pathogen identified, comprising 31.3% of cases. These pathogens in SA are associated with elevated mortality rates and profound joint dysfunction [33]. Furthermore, *S. aureus* infections exhibit higher rates of cellulitis, abscess formation, and increased morbidity, leading to interventions such as fusion, amputation, or prosthetic joint replacement. Notably, drug-resistant strains, including *MRSA*, are becoming more prevalent, especially in intravenous (IV) drug users, with vancomycin-resistant strains observed in patients with recurrent healthcare-associated infections [48].

Other studies identified MRSA infections were frequently associated with oligoarticular arthritis, while group B streptococci were more prevalent in cases of oligoarticular arthritis compared to monoarticular septic arthritis [29, 49]. In our study, the three *MRSA* cases demonstrated oligoarticular arthritis. Moreover, *streptococci* cases exhibited a predominance in monoarticular cases (7 vs. 3), and a significant pattern was observed in *S. aureus* cases.

Understanding the microbial landscape of SA is crucial for tailoring effective treatment strategies, particularly in the context of emerging drug-resistant strains and the varied clinical manifestations associated with different pathogens. Further research is warranted to explore the evolving epidemiology of SA pathogens and their implications for patient outcomes.

Certain limitations should be acknowledged when interpreting the findings of this study. It is a single-center retrospective design, conducted in a German university hospital, may restrict the generalizability of findings to diverse populations. There is a potential for inherent selection bias as the study exclusively involves patients from an academic medical center, which could influence the observed severity and comorbidity profiles. The sample size of 192 patients, while relatively large compared to other studies, may still constrain the statistical robustness and broader generalizability of the findings. Diagnostic challenges in identifying SA, despite efforts to ensure accuracy, could lead to misclassifications or underdiagnoses. Dependence on electronic health records introduces the potential for data gaps or incompleteness, thereby influencing the precision of comorbidity assessments. The minimum follow-up period of 24 months may not adequately capture long-term outcomes, and statistical methods, while robust, lack specific details. Another limitation is that the SIRS criteria for patient assessment were employed, which have faced scrutiny regarding their effectiveness in recent years. Current guidelines suggest using more precise tools, such as qSOFA and SOFA scores, to evaluate sepsis severity for improved accuracy.

## Conclusion

A vulnerable patient group demonstrated a notably elevated mortality rate. Understanding the intricate interplay of factors contributing to mortality is paramount. Analyzing the specific risk factors unique to each patient, such as existing health conditions, is crucial in effectively addressing and treating SA. Our results emphasize the critical need for careful monitoring, particularly among SA patients with chronic organ diseases, prompt identification and handling of sepsis. These measures collectively aim to improve the chances of survival for this vulnerable group of patients.

## Data Availability

The datasets used and/or analyzed during the current study are available from the corresponding author on reasonable request.
